# Lower Health Risks of Potentially Toxic Metals after Transplantation of Aquacultural Farmed Mussels from a Polluted Site to Unpolluted Sites: A Biomonitoring Study in the Straits of Johore

**DOI:** 10.3390/foods12101964

**Published:** 2023-05-11

**Authors:** Chee Kong Yap, Khalid Awadh Al-Mutairi

**Affiliations:** 1 Department of Biology, Faculty of Science, Universiti Putra Malaysia (UPM), Serdang 43400, Malaysia; 2 Department of Biology, Faculty of Science, University of Tabuk, Tabuk 741, Saudi Arabia; kmutairi@ut.edu.sa

**Keywords:** risks, metals, transplantation, mussels

## Abstract

The present field-based study aimed to determine the levels of six potentially toxic metals (PTM)s (Cd, Cu, Fe, Ni, Pb, and Zn determined using a flame atomic-absorption spectrophotometer) using transplanted green-lipped mussel *Perna viridis* from a polluted site at Kampung Pasir Puteh (KPP) to unpolluted sites at Kampung Sungai Melayu (KSM) and Sungai Belungkor (SB) in the Johore Straits (SOJ), and to estimate the human health risks of the PTMs after the depuration periods. Interestingly, after 10 weeks of depuration in the two unpolluted sites, there were 55.6–88.4% and 51.3–91.7% reductions of the six PTMs after transplantation from KPP to SB and KSM, respectively. Lower risks of health assessments were recorded and judged on the present findings of significantly (*p* < 0.05) lower levels of safety guidelines, significantly (*p* < 0.05) lower values of target hazard quotient, and significantly (*p* < 0.05) lower values of estimated weekly intake, of all the six PTMs after 10 weeks of depuration of the transplanted polluted mussels to the two unpolluted sites in the SOJ. Thus, further reducing the noncarcinogenic risks of the PTMs to the consumers. From an aquacultural point of view, this depuration technique can be recommended to reduce the health risks of PTMs to mussel consumers.

## 1. Introduction

The aquaculture production of mussels in Malaysia in 2001 [1] and 2021 [2] were 6880.1 tonnes and 1472.51 tonnes, respectively. This indicated a decrease of 78.6% in mussel production from 2001 to 2021. The Johore’s state covered most of production (96.2% or 6616.06 tonnes in 2001; 92.6% or 1363.35 tonnes in 2021). This showed that there was a decrease of 79.4% in the mussel production in Johore from 2001 to 2021 [1,2]. In contrast, the wholesale value of aquaculture mussels in Malaysia were RM 3,299,800 and RM 7,415,630 in 2001 [1] and 2021 [2], respectively. This indicated that there was an increase of 124.7% wholesale value from 2001 to 2021. The state of Johore covered a majority of the mussel wholesale value (88.2% or RM 2,911,070 in 2001; 90.3% or RM 6,698,460 in 2021). This showed that there was an increase of 130.1% in the mussel wholesale value in Johore from 2001 to 2021 [1,2]. Similarly, the mussel aquaculture area in Malaysia were 79,052 m^2^ and 206,902 m^2^ in 2001 [1] and 2021 [2], respectively. This indicated that there was an increase of 161.7% mussel aquaculture area from 2001 to 2021. The state of Johore covered a majority of the mussel aquaculture area (71.4% or 56,465 m^2^ in 2001; 95.5% or 197,672 in 2021). This showed that there was an increase of 250% in the mussel aquaculture area in Johore from 2001 to 2021 [1,2]. Prices (US dollar) for Malaysia mussels (per kilogram) have been reported to be $7.00, US$2.60, and $2.35, in 2017, 2018 and 2019, respectively [3]. This signifies the importance of mussel markets in Malaysia. In summary, even though mussel production and supply has decreased in the last two decades in the Straits of Johore (SOJ), the mussel wholesale values and mussel aquaculture area have increased significantly from 2001 to 2021. Most interestingly, the mussel aquaculture became very important in the seafood sector in the SOJ. Hence, innovation in the mussel product quality to lower the risk to consumers in this seafood sector is highly necessary. A new mussel aquaculture farm has recently been established in Tanjung Langsat in the SOJ, and it is anticipated that it will begin producing its first batch of mussels in January 2023 [4]. The new farm is a component of the initiative by the Malaysian Fisheries Development Authority and the Johor Fisheries Department to expand the production of mussels in the state of Johor.

Marine mussels are a suitable option for transplantation experiments since they are reliable and affordable [5,6]. They also satisfy several requirements, such as leading a sedentary lifestyle, having sufficient tissue for metal analysis, consuming suspensions, tolerating high concentrations of heavy metals, and being prone to bioaccumulate and amplifying such metals [6,7,8,9]. This study focuses on the human health risk of potentially toxic metals (PTMs) because the mussels are commercially cultured and sold to consumers. This study is also part of the Mussel Watch program because the marine mussels have fulfilled most of the important criteria as a good biomonitor, including being of public concern (because they are also seafood delicacy to the consumers) [5,6,7,8]. In actuality, many important biological processes related to metal exposures have traditionally been explored in laboratory rather than through outdoor investigations [5,10]. Mussel transplantation is a more feasible solution to monitor the accumulation of metal in mussels over time. Shi et al. [11] noted that the current study demonstrated the suitability of transplanted green-lipped mussels from developed coastal regions in eastern Guangdong (China) for assessing heavy metal pollution, notably for Cd, Cu, and Zn.

The usage of transplanted mussels in coastal locations has been the subject of several reports. This is due to their numerous benefits and their effectiveness in biomonitoring studies. Examples include *Scrobicularia plana* in a coastal lagoon (Ria de Aveiro, Portugal) [12], *Mytilus galloprovincialis* in the Mediterranean Sea [13,14], *Perna perna* in Brazil *Crenomytilus grayanus*, *Mytilus edulis* in the Aegean Sea [15], *M. edulis* in the Gulf of Finland [16], and *M. edulis* in South Greenland [17]. All of the aforementioned findings made it abundantly evident that bivalve field transplantations are crucial for developing the scientific framework for investigating the effects of pollution on aquatic ecosystems. By using transplanted mussels, external and internal influences that may skew data comparisons, such as seasonal variation, size, and age, are lessened. [10,18]. The monitoring locations in transplantation studies can be chosen on their own without taking into account the existence of natural populations [10,18,19,20]. The present study looked into the possibility of the metal-contaminated mussel populations being depurated in relatively clean coastal waters using the transplantation method. This could provide an answer to the sustainable supply of high demand of mussel seafood even though they are cultured in polluted waters, as long as they were transplanted to unpolluted wasters before harvesting for human consumption.

Therefore, the aim of this paper was to determine the levels of Cd, Cu, Fe, Ni, Pb, and Zn (determined using flame atomic absorption spectrophotometer) using transplanted *P. viridis* from a polluted site at Kampung Pasir Puteh (KPP) to unpolluted sites at Sungai Belungkor (SB) and Kampung Sungai Melayu (KSM) in the SOJ, and to estimate the human health risks of the PTMs after depuration periods.

## 2. Materials and Methods

### 2.1. Study Sites of Transplantations

**Figure 1:**
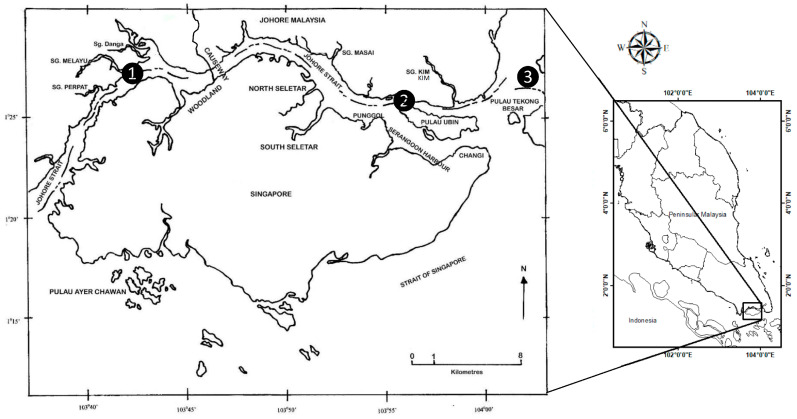
Sampling site at Kampung Pasir Puteh (❷), and mussel transplantation sites at Kg. Sungai Melayu (❶) and Sungai Belungkor (❸) in the Straits of Johore in the present study.

The KPP (Figure 1) is considered a polluted site impacted by numerous human activities due to two important facts. First, there are observable human activities in the surroundings of KPP, including the marinas, petrol-chemical plants, super oil tankers, restaurants, and receiving urban domestic wastes. Second, KPP is near the industrial site in the southern part of the Peninsular Malaysia-Pasir Gudang Industrial Area [21]. When comparing our unpublished sediment data with those reported by Yap et al. [5] for the same sampling location, the sediment data acquired from KPP in this study were typically higher than KSM. This indicated that KPP was more heavily polluted with metals than KSM [22,23].

The contamination by Zn, Cd, Cu, and Pb at KPP in the SOJ had been previously reported by Yap et al. [5,21,23]. Large shipyard maintenance and fossil fuel-fired power plants, construction facilities, and shipping dock operations can all be found at KPP, which is situated in the eastern section of causeway and is considered to be a highly polluted location [23,24,25]. On the contrary, KSM is thought to be impacted by few human operations, with fish and mussel aquaculture being the only activities present there [21]. Therefore, KSM and SB are regarded as low-human-activity sites and pristine areas in the SOJ, respectively [26].

### 2.2. Mussel Population Transplants

On 28 November 2009, about 200 *P. viridis* individuals were collected from KPP and transferred to SB and KSM in the Straits of Johore. Following the mussel collection, the entire cluster was washed three times in seawater to remove any apparent debris on the mussel shells. The mussels were then separated into 40 distinct subgroups at random, and each subgroup was put in a polyethylene cage that was 20 cm × 15 cm × 18 cm in size and water was allowed to circulate through it. The mussels were sampled before the experiment began (W1), after 2 (W2), 6 (W6), and 10 (10 W) weeks of depuration. Before being brought back to the lab in an ice compartment at 10 °C, the collected mussels were washed with saltwater. The variation of shell length (cm), shell width (cm), shell height (cm), and growth rate (cm/week) are presented in Table S1.

The dried samples were digested in concentrated nitric acid (AnalaR grade, BDH 69%) in a hot-block digestor for 1 h at a low temperature (40 °C). After that, they were raised to 140 °C for 3 h [5]. After being digested, the samples were diluted in 40 mL of double-distilled water. The sample was then filtered using Whatman No. 1 (filter speed: medium) filter paper (GE Healthcare Whatman^TM^, Chicago, USA), and the filtrates were kept until the metal determination in pill boxes that had been acid washed. Using a Perkin-Elmer Model AAnalyst 800 air-acetylene flame atomic-absorption spectrophotometer (Perkin Elmer LLC., Shelton, CT, USA), the prepared samples were determined for Cd, Cu, Fe, Ni, Pb, and Zn.

### 2.3. Quality Monitoring and Assurance

All utilized glassware and equipment were acid cleaned to prevent contamination, and the accuracy of the analysis was verified using blanks. For the purpose of validating the data, the Certified Reference Material (CRM) for dogfish liver (DOLT-3, National Research Council Canada, Ottawa, ON, Canada) and mussel tissue (NIST 2976, IAEA, Monaco) were compared to the recoveries for each, as shown in Table S2.

### 2.4. Data Treatment

#### Human Health Risk Assessments

The PTMs data on a dry weight (dw) basis were converted into a wet weight (ww) one for human health risk assessment (HHRA) using a conversion factor of 0.17 for *P. viridis* [27]. Three assessments were conducted to determine the HHRA resulting from ingestion of the mussels, namely:

(a) Direct comparisons with seafood safety guidelines

Only the maximum permitted Ni limits (MPL), sometimes referred to as the action levels (80 mg/kg WW) for molluscan shellfish, were included in the comparison [28]. The comparison for Cd was based on the Cd MPLs (1–2 mg/kg ww) of the United Nation’s Food and Agriculture Organization (FAO) compilation of the legal limits by FAO (which are equivalent to MPL) for Cd in fish/fish products/shellfish in three developed countries (New Zealand, UK, and Australia [29]), and the MPL (2.00 mg/kg w) set by the Codex Alimentarius Commission in marine bivalves, including mussels [30].

For Pb, the comparison was based on the Pb MPLs suggested by the commission regulation of the European Union (1.50 mg/kg ww; EC [31], US Food and Drug Admin-istration (1.70 mg/kg ww; US Food and Drugs Administration/Interstate Shellfish Sanitation Conference (USFDA/ISSC) [28], ANZFSC (Australian New Zealand Food Standards Code) (2.00 mg/kg ww; [32]), Malaysian Food Regulations 1985 (2.00 mg/kg ww; [33]), and the legal limits of Pb (2–10 mg/kg ww) as compiled by FAO [29], based on those of New Zealand, the UK, and Australia. 

In terms of Cu, the comparisons were based on the Cu MPLs recommended by the Malaysian Food Regulations (30 mg/kg ww; [33]), and FAO [29] was based on the nations of New Zealand, the UK, and Australia, with the range of the legal limits of Cu as 20–70 mg/kg ww. The comparisons for Zn were based on MPLs recommended by FAO [29], which ranged from 40 to 150 mg/kg ww based on New Zealand, the UK, and Australia, as well as the Malaysian Food Regulations (100 mg/kg ww; [33]). However, before 2015, the World Health Organization (WHO), US Food and Drugs Administration (USFDA), FAO, and other nations hardly imposed any Fe maximum limits. Before now, JECFA [34], based on the opinions of a global group of experts, concluded that there was still some doubt over the maximum level of Fe that may be tolerated. As a result, it is impossible to compare MPLs to the available Fe data.

(b) Target hazard quotient

The estimated daily intake (EDI) must first be computed before determining the target hazard quotient (THQ). EDI calculates the specific metal intake by employing body weight (bw) and the rate of mussel consumption. It was determined according to Equation (1):

EDI = (Mc × CR)/bw(1)

where CR = average daily consumption rates of mussels for Malaysian adults based on 2675 respondents (Malay: 76.9%; Chinese: 14.7%; and India: 8.4%); Mc = metal content in the samples (mg/kg) on a ww basis; [35]. According to Nurul Izzah et al. [35], the typical adult population of Malaysia has a bw of 62 kg, and the high-level consumer rate is expected to be two times that amount.

The THQ was later determined using Equation (2):

THQ = EDI/ORD(2)

where ORD = oral reference dose.

The oral reference dose (ORD) estimates a contaminant’s lifetime daily consumption that is unlikely to result in adverse health effects [36]. The ORD values (µg/kg/day) used in this study were: Cd: 1.00; Cu: 40.0; Ni: 20.0; Fe: 700; Pb: 3.50, and Zn: 300, provided by the USEPA’s regional screening level [36].

(c) Comparisons between estimated weekly intake (EWI) and provisional tolerable weekly intake (PTWI):

The Joint FAO/WHO Expert Committee on Food Additives created the provisional tolerable weekly intake (PTWI) [37]. Calculating weekly metal exposure levels and comparing the results to the relevant recommended PTWI levels, the danger that food intake caused to people’s health was assessed. The quantity of a substance that is thought to be present in food or drinking water and that may be consumed without significantly harming health over a lifetime is known as the PTWI, and it is expressed in mg/kg of body weight [38]. As a result, it was determined how much seafood from this study exceeded the PTWI limitations. 

Table S3 lists the ORD values (g/kg bw/day) and PTWI values (mg/kg bw/week) for Cd, Cu, Ni, Fe, Pb, and Zn that were employed in the current investigation. The values of the Zn PTWI were computed using the JECFA-based provisional tolerable daily intake (PTDI) [39,40]. The preliminary maximum tolerated daily intake (PMTDI) values for Fe were recalculated based on JECFA [34,40], and those of PTWI were recalculated for Cu using PMTDI and JECFA [39,40]. The values of the Ni PTWI were computed using the EFSA-recommended Tolerable Daily Intake (TDI) [41], and those of PTWI for Pb using TDI and JECFA [42]. From the provisional tolerable monthly intake (PTMI) based on JECFA [40,42], the values of PTWI for Cd were adjusted. The average adult weight in Malaysia is 62 kg, so maintaining that weight requires 5642 g/week of Ni, 1302 g/week of Pb, 361.5 g/week of Cd, 217,000 g/week of Cu, and 347,200 g/week of Fe. 

To calculate the risk of exposure through ingestion, the value of the estimated weekly intake (EWI) for each metal in *P. viridis* was calculated as follows:

EWI = EDI × 7(3)

where EDI = estimated daily intake as calculated in Equation (1), multiplied by seven because there are seven days in a week.

### 2.5. Statistics Analysis

All graphical bar charts were produced using KaleidaGraph (Version 3.08, Sygnergy Software, Eden Prairie, MN, USA). Curve fit employed an exponential regression to describe the relationship between the metal levels in mussel soft tissues at 4, 6, and 10 weeks after depuration. The exponential decay model was conceptually acceptable and rational since it generated the best plot with a decay constant (with an R-value) for the relationships that satisfied the objective of this investigation, namely the reduction in metal levels to the potential pre-exposure metal levels [43].

StatSoft Inc.’s STATISTICA version 8.0 (Tulsa, OK, USA) for Windows was used to perform the statistical analysis. For each of the six metals, a one-way ANOVA was used to compare the means of the metals [43].

## 3. Results

The mean concentrations and their general data are presented in Table 1. 

**Figure 2:**
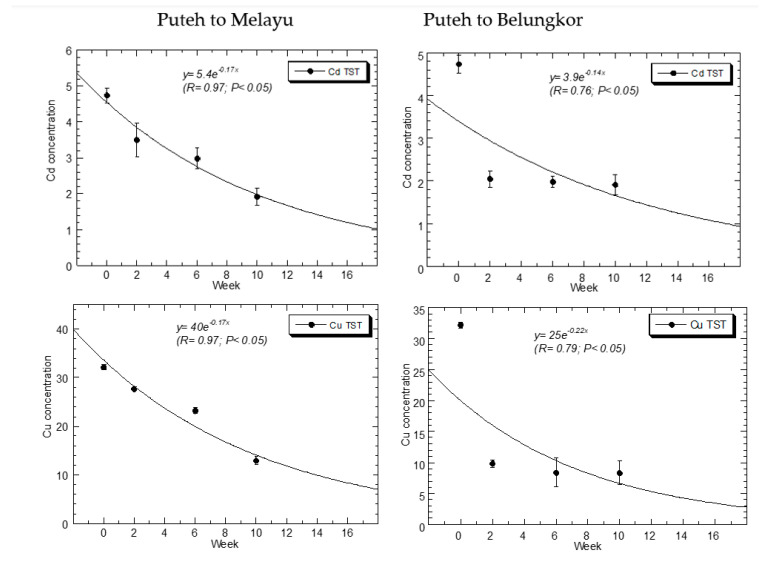

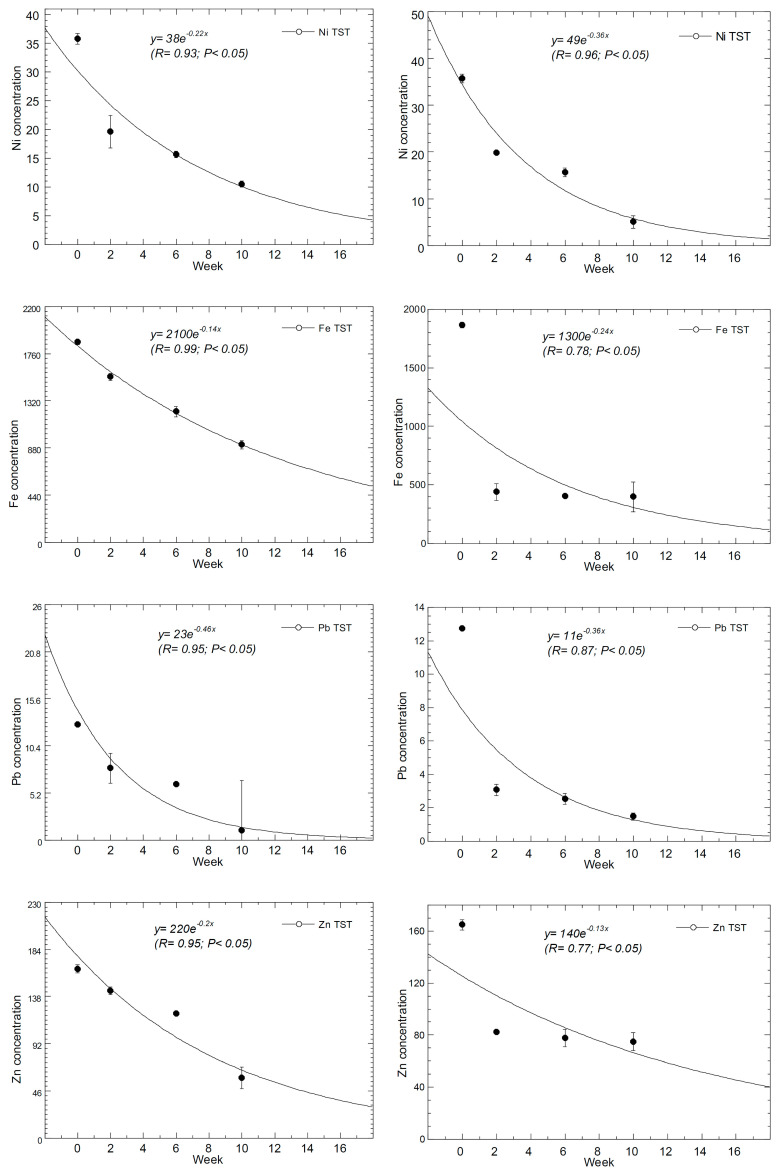
Depuration of Cd, Cu, Fe, Ni, Pb, and Zn concentrations (mg/kg dry weight) in the total soft tissues (TST) of *Perna viridis* transplanted from Kg. Pasir Puteh to Kg. Sungai Melayu (Puteh to Melayu; (**left**)), and to Sungai Belungkor (Puteh to Belungkor; (**right**)). Curve fits are based on exponential equations.

The Ni in Figure 2 used an exponential decay model to estimate the best plot with a decay constant (λ) of 0.22 (R = 0.93; *p* < 0.05) to summarize the negative relationship between Ni levels and weeks after transplantation from KPP to KSM. Similarly, a higher decay constant (λ) of 0.36 (R = 0.96; *p* < 0.05) to summarize the negative relationship between Ni levels and weeks after transplantation from KPP to SB. This exponential regression can model a situation of a faster decrement of Ni levels in the experimental transplanted KPP to SB than KPP to KSM.

### 3.1. Ni

#### 3.1.1. Comparison with Reported Studies and Food Safety Guidelines of Nickel

After 10 weeks of transplantation, the percentages of Ni reductions were 85.9% and 70.6% from KPP to SB, and KPP to KSM, respectively (Table 1 and Table 2; Figure 2).

The present Ni ranges (5.05–35.8 mg/kg dry weight; 0.86–6.09 mg/kg wet weight) were within those (1.94 to 114 mg/kg dw) reported by Yap et al. [44] in 40 populations of *P. viridis* from the coastal waters of Peninsular Malaysia. The current ranges of Ni levels were still greater than those found in the coastal waters of Singapore (3.80–13.0 mg/kg dw; [22]), Karnataka (0.02–1.26 mg/kg ww; [45]), Hong Kong (1.70–16.5 mg/kg dw; [46]), and Trinidad and Venezuela (0.22–1.30 mg/kg ww; [47]). Nicholson and Szefer [48] showed that Ni concentrations ranged from 2.84 to 13.4 mg/kg dw in the soft tissues of *P. viridis* in the contaminated and unpolluted areas of Hong Kong coastal waters. Fung et al. [49] reported the concentrations (mg/kg dw) in *P. viridis* collected from Minjiang and Jiulongjiang (east coast of China) as Ni (2.97–4.78). Chinnadurai et al. [50] reported that the Ni concentrations (0.46–0.79 mg/kg ww) in *P. viridis* were collected from the Vembanad Estuary, Kerala (India). As a result, the current Ni values are greater than those that were reported in the literature for the same mussel species.

In the literature, MPLs for Ni are uncommon [51]. However, the US Food and Drug Administration [28] set an action level (Ni: 80 mg/kg ww), which is the only Ni MPL currently available. Consequently, the Ni concentrations were all far below the Ni action limit in *P. viridis* populations.

#### 3.1.2. Comparisons of Nickel Target Hazard Quotients

The values of Ni EDI and Ni THQ in the mussels are summarized in Table 3. After 10 weeks of depuration, the values of EDI decreased from 3.92 to 1.15 in KPP to KSM. The Ni EDI values decreased from 3.92 to 0.55 in KPP to SB. After 10 weeks of depuration, the values of THQ decreased from 0.20 to 0.06 in KPP to KSM. The THQ values decreased from 0.20 to 0.03 in KPP to SB. All the THQ values were below 1.00, indicating that there was no noncarcinogenic risk of Ni in the transplanted mussels to SB and KSM. The Ni human health risk was significantly (*p* < 0.05) lower after the mussels were transplanted to SB and KSM from KPP.

#### 3.1.3. Comparisons between Estimated Weekly Intake (EWI) and Provisional Tolerable Weekly Intake (PTWI)

The values of Ni EWI in *P. viridis* and Ni PTWI in the mussels are summarized in Table 3. After 10 weeks of depuration, the values of Ni EWI decreased from 27.5 to 8.07 µg/week in KPP to KSM with a 70.7% reduction. In comparison to the Ni PTWI, the Ni EWI at week 0 contributed 0.487% and further reduced to 0.143% after 10 weeks of depuration. The Ni EWI values decreased from 27.5 to 3.88 µg/week in KPP to SB with an 85.9% reduction. In comparison to the Ni PTWI, the Ni EWI at week 0 contributed 0.487% and was further reduced to 0.069% after 10 weeks of depuration. The PTWI figure (5642 g/week) was clearly higher than any of the EWI values. According to the FAO/WHO JECFA recommendations, eating mussels was not deemed to be harmful to consumers due to nickel.

A tolerated daily intake (TDI) of 13.0 g/kg bw has been established by EFSA [41]. As a result, Ni’s PTWI is 13.0 μg/kg bw/week (91 mg/kg bw/week). Therefore, the Ni PTWI for a 62 kg adult in Malaysia is comparable to 5642 g/week.

Ni has been specifically examined in marine mussels for its uptake and depuration, absorption and metabolic responses, and bioaccumulation [18]. These results highlight the importance of Ni and its exceptional potential for being highly accumulative in marine mussels that are used to produce seafood for human consumption. Ni is a prevalent metal in nature, but its value as a trace element for both animals and people has not yet been recognised [52]. Municipal and industrial waste, the use of liquid and solid fuels, and industry all have the potential to contaminate the environment with Ni.

All types of soil, meteorites, and volcanic eruptions contain nickel. Nickel is most frequently coupled with oxygen or sulfur in the atmosphere to form oxides or sulfides in the Earth’s crust [53]. There is no question that exposure to nickel at work or the pervasive usage of Ni in a variety of sectors has a harmful impact on people’s health. Batteries, alloys, and stainless steel are only a few products that employ nickel and its derivatives [54]. 

### 3.2. Cd

#### 3.2.1. Cd Safety Guidelines

After 10 weeks of transplantation, the percentages of Cd reductions were 59.7% and 59.5% from KPP to SB and from KPP to KSM, respectively (Table 1 and Table 2; Figure 2).

With a decay constant (λ) of 0.17 (R = 0.97; *p* < 0.05) to summarize the adverse correlation between Cd levels and weeks following transplantation from KPP to KSM, the Cd in Figure 2 employed an exponential decay model. The negative correlation between Cd levels and weeks following transplanttion from KPP to SB is also summarized by a comparative decay constant (λ) of 0.14 (R = 0.76; *p* < 0.05). The comparative decline in Cd levels between experimentally transplanted KPP to SB and KPP to KSM may be modeled using this exponential regression.

The present Cd ranges (1.92–4.74 mg/kg dry weight) were wider than those (0.35–3.15 mg/kg dw) reported by Yap et al. [44] in 40 populations of *P. viridis* from the coastal waters of Peninsular Malaysia. The present Cd ranges were comparatively higher (especially the maximum level) than those from Singapore (>0.01–0.20 mg/kg dw; [22]) and Peninsular Malaysia (0.25–1.25 mg/kg dw; [23]). However, they are lower than those (0.70–2.02 mg/kg ww) reported for the coastal waters of Karnataka (India), by Sasikumar et al. [45]; de Astudillo et al. [47] reported the metal levels in *P. viridis* collected from Trinidad and Venezuela were Cd (0.01–0.61 mg/kg ww). Nicholson and Szefer [48] showed that Cd concentrations ranged from 1.02 to 5.40 mg/kg dw in the soft tissues of *P. viridis* in contaminated and unpolluted areas of Hong Kong coastal waters. Fung et al. [49] reported the concentrations (mg/kg dw) in *P. viridis* collected from Minjiang and Jiulongjiang (east coast of China) as Cd (0.48–1.10). Aktar et al. [55] reported the *P. viridis* collected from Cox’s Bazar (Bangladesh) was 0.04 to 0.08 mg/kg dw for Cd. Kamaruzzaman et al. [56] reported Cd (0.58 mg/kg dw) in *P. viridis* collected from the Muar estuary. Mussels collected in 2009 from the Pekan River estuary (Pahang) coastal waters were reported as Cd (0.30 mg/kg dw) [57]. In mussels collected from Sungai Masai in the SOJ, Sheng et al. [58] reported that the concentration (mg/kg dw) was Cd (0.14). Mamat et al. [59] reported that the *P. viridis* collected from KPP was 9.10–13.0 mg/kg dw for Cd. Soegianto et al. [60] reported that the mussels collected from the East Java coast contained Cd (0.005–2.47 mg/kg ww). Montojo et al. [61] reported the Cd concentrations in *P. viridis* collected from Manila Bay as 0.0043–0.0338 mg/kg ww. All the above comparisons show that the present Cd ranges were within or higher than most of the reported studies.

The current Cd ranges (0.33–0.81 mg/kg wet weight) did not exceed all the Cd safety guidelines, which were based on the Cd MPL (1.00 mg/kg ww) set by MFR [33]; 2.00 mg/kg ww, set by the Codex Alimentarius Commission in marine bivalves (including mussels) [30]; and the commission regulation of the European Union [31]. The MPLs (1–2 mg/kg ww) of the FAO compilation of the legal limits (which were equivalent to MPL) of Cd in fish/fish products/shellfish from the UK, New Zealand, and Australia [29] were the MPL (4.00 mg/kg ww) set by the USFDA/ISSC [28], and the MPL (2.00 mg/kg ww) set by ANZFSC [32].

#### 3.2.2. Comparisons of Cadmium Target Hazard Quotients

Values of Cd EDI and Cd THQ in mussels are summarized in Table 3. After 10 weeks of depuration, both the values of EDI and THQ decreased from 0.52 to 0.21, in both KPP to KSM and KPP to SB. All the Cd THQ values were below 1.00, indicating that there was no noncarcinogenic risk of Cd in the mussels transplanted to SB and KSM. The Cd human health risk was significantly (*p* < 0.05) lower after the mussels were transplanted to SB and KSM from KPP.

#### 3.2.3. Comparisons between Cadmium Estimated Weekly Intake (EWI) and Provisional Tolerable Weekly Intake (PTWI)

The values of Cd EWI in *P. viridis* and Cd PTWI in the mussels are summarized in Table 3. After 10 weeks of depuration, the values of Cd EWI decreased from 3.64 to 1.47 µg/week in both KPP to KSM and KPP to SB, with a 59.6% reduction. In comparison to the Cd PTWI, the Cd EWI at week 0 contributed 1.01% and further reduced to 0.407% after 10 weeks of depuration. Clearly, all of the EWI values were significantly lower than the Cd PTWI value (361.5 µg/week). Therefore, the mussel ingestion was not considered to have adverse effects of Cd on consumers based on the FAO/WHO JECFA guidelines.

According to Horiguchi et al. [62], who based their findings on daily consumption of rice, female Japanese farmers who had consumed foods containing Cd at a level close to the current PTWI did not show increased development of renal tubular dysfunction in comparison to other groups with lower Cd exposure. Because industrial and agricultural activities have dispersed Cd across the environment, the impact of Cd contamination on public health is a significant problem. According to Oberdorster [63], breathing in contaminated air is the principal method of people’s exposure to environmental Cd. Consuming Cd-contaminated food, such as shellfish, is still a prevalent method. Female farmers who lived in Cd-polluted areas were the subject of an investigation by Horiguchi et al. [64] into Cd’s exposure, accumulation, renal consequences, and the relationship between age and Cd effects. They deduced that rice consumption contributed to excessive Cd buildup and resulted in a deterioration of renal function in older women living in polluted surroundings. Itai Itai Disease, the most severe type of chronic Cd poisoning, was originally discovered among female farmers in Japan’s Jinzu River Basin, which has high levels of Cd contamination and is connected to renal anemia and osteomalacia [65].

According to a study by Jovic and Stankovic [66], the amount of mussels that may be consumed without exceeding the JECFA limit for Cd is highest in Croatia (0.57 kg per week; Cd mean: 0.61 mg/kg ww), where the highest Cd mean concentrations were reported, and lowest in Albania (0.08 mg/kg ww). Comparatively, Jovic and Stankovic [66] discovered that, based on the observed Cd content in mussels from the Adriatic Sea, the projected weekly Cd intake for high-level mollusks consumers was 0.076 mg Cd/person/week and 0.15 mg Cd/person/week. While Cd is a non-essential element for living things, there should not be great public concern, because the Cd ranges in mussel soft tissues are lower than all of the Cd MPLs [67].

### 3.3. Pb

#### 3.3.1. Pb Safety Guidelines and Comparison with Reported Studies

After 10 weeks of transplantation, the percentages of Pb reductions were 88.4% and 91.7% from KPP to SB, and KPP to KSM, respectively (Table 1 and Table 2; Figure 2).

The Pb in Figure 2 used an exponential decay model to estimate the best plot with a decay constant (λ) of 0.46 (R = 0.95; *p* < 0.05) to summarize the negative relationship between Pb levels and weeks after transplantation from KPP to KSM. Similarly, a lower decay constant (λ) of 0.36 (R = 0.87; *p* < 0.05) summarizes the negative relationship between Pb levels and weeks after transplantation from KPP to SB. This exponential regression can model a situation of a lower decrement of Pb levels in the experimental transplanted KPP to SB than in KPP to KSM.

The present Pb ranges (1.06–12.7 mg/kg dry weight; 0.18–2.16 mg/kg wet weight) were within those (1.57–61.04 mg/kg dw) reported by Yap et al. [44] in 40 populations of *P. viridis* from the coastal waters of Peninsular Malaysia. The present Pb ranges after 10 weeks of depuration were also lower than those from Hong Kong coastal waters (2.00–20.0 mg/kg dw; [46]), Peninsular Malaysia (1.80–8.76 mg/kg dw; [23]), Singapore (BDL–7.20 mg/kg dw; [22]), and the coastal waters of Karnataka, Southwest Coast of India (BDL–1.90 mg/kg ww; [45]). Nicholson and Szefer [48] showed that Pb concentrations ranged from 3.10 to 6.98 mg/kg dw in the soft tissues of *P. viridis* in contaminated and unpolluted areas of Hong Kong coastal waters. Fung et al. [49] reported the concentrations (mg/kg dw) in *P. viridis* collected from Minjiang and Jiulongjiang (east coast of China) as Pb (1.23–2.93). Chinnadurai et al. [50] reported that the Pb concentrations were (0.40–2.24 mg/kg ww) in the *P. viridis* collected from Vembanad Estuary, Kerala (India). Aktar et al. [55] reported the *P. viridis* collected from Cox’s Bazar (Bangladesh) was 0.19 to 0.75 mg/kg dw for Pb. Kamaruzzaman et al. [62] reported Pb (2.28 mg/kg dw) in *P. viridis* collected from the Muar estuary. Mussels collected in 2009 from the Pekan River estuary (Pahang) coastal waters were reported as Pb (0.47 mg/kg dw) [57]. Mussels collected from Sungai Masai in the SOJ, Sheng et al. [58] reported that the concentration (mg/kg dw) was Pb (0.41). Mamat et al. [59] reported the *P. viridis* collected from KPP was 25.1–38.6 mg/kg dw for Pb. Soegianto et al. [60] reported that the mussels collected from the East Java coast contained Pb (0.06–3.50 mg/kg ww). Montojo et al. [61] reported the Pb concentrations in *P. viridis* collected from Manila Bay as 0.0480 mg/kg ww. Thus, the present Pb ranges are within or lower than those reported in the same mussel species in the literature. Riani et al. [68] examined the effect of heavy metal pollution on green mussels cultured in the Muara Kamal Waters, Jakarta Bay over seven months. Based on the heavy metal concentrations in their bodies, the main cause of malformations in green mussels was suspected to be contamination by three metals, including Pb. They concluded that the interaction between nitrogen compounds, phosphate, turbidity, salinity, and pH, as well as the heavy metals in the water, determined the green mussel abnormality.

The Pb levels after 10 weeks of transplantation to SB and KSM were considerably lower than all the Pb safety guidelines [28,29,31,32,33].

#### 3.3.2. Comparisons of Lead Target Hazard Quotients

The values of Pb EDI and Pb THQ in the mussels are summarized in Table 3. After 10 weeks of depuration, the values of EDI decreased from 1.40 to 0.12 in KPP to KSM. The Pb EDI values decreased from 1.40 to 0.16 in KPP to SB. After 10 weeks of depuration, the values of Pb THQ decreased from 0.399 to 0.033 in KPP to KSM. The Pb THQ values decreased from 0.399 to 0.046 in KPP to SB. All the THQ values were below 1.00, indicating that there was no noncarcinogenic risk of Pb in the transplanted mussels to SB and KSM. In fact, the depuration transplantation study in both sites had further reduced the Pb risk to consumers. The Pb human health risk was significantly (*p* < 0.05) lower after the mussels were transplanted to SB and KSM from KPP.

#### 3.3.3. Comparisons between Pb Estimated Weekly Intake (EWI) and Provisional Tolerable Weekly Intake (PTWI)

The values of Pb EWI in *P. viridis* and Pb PTWI in the mussels are summarized in Table 3. After 10 weeks of depuration, the values of Pb EWI decreased from 9.78 to 0.81 µg/week in KPP to KSM with 91.7% reduction. In comparison to the Pb PTWI, the Pb EWI at week 0 contributed 0.751% and further reduced to 0.062% after 10 weeks of depuration. The Pb EWI values decreased from 9.78 to 1.14 µg/week in KPP to SB with 88.3% reduction. In comparison to the Pb PTWI, the Pb EWI at week 0 contributed 0.751% and further reduced to 0.088% after 10 weeks of depuration. Clearly, all of the EWI values were significantly lower than the Pb PTWI value (1302 µg/week). Therefore, the mussel consumption was not considered to have adverse effects of Pb to consumers based on the FAO/WHO JECFA guidelines [42].

Italy and Slovenia are the countries with the highest mean Pb contents (1.01 mg/kg ww), but the lowest and highest weekly consumption rates of mussels (0.26 kg; Pb mean: 0.34 mg/kg ww). Comparatively, Jovic and Stankovic [66] found that the estimated weekly intake of Pb for frequent consumers of mollusks was 0.13 mg/person/week and 0.25 mg/person/week, based on the Pb contents observed in mussels from the Adriatic Sea. 

Pb is a non-essential element that has been connected to, among other health problems, neurotoxicity and nephrotoxicity [69]. Due to its potential to accumulate in significant amounts in the human body and the fact that it is not known to serve any biological purpose, this hazardous metal may pose a substantial threat to public health [66]. The body’s mineralizing mechanisms and soft tissues, such as the blood, liver, and kidneys (bones and teeth), carry lead. Children who experience Pb poisoning may have lower IQs and cardiovascular problems [70]. 

### 3.4. Cu

#### 3.4.1. Cu Safety Guidelines and Comparison with Reported Studies

After 10 weeks of transplantation, the percentages of Cu reductions were 74.2% and 59.8% from KPP to SB, and KPP to KSM, respectively (Table 1 and Table 2; Figure 2).

The Cu in Figure 2 used an exponential decay model to estimate the best plot with a decay constant (λ) of 0.17 (R = 0.97; *p* < 0.05) to summarize the negative relationship between Cu levels and weeks after transplantation from KPP to KSM. Similarly, a higher decay constant (λ) of 0.22 (R = 0.79; *p* < 0.05) summarizes the negative relationship between Cu levels and weeks after transplantation from KPP to SB. This exponential regression can model a situation of a faster decrement of Cu levels in the experimental transplanted KPP to SB than in KPP to KSM.

The present Cu ranges were well below all the Cu safety guidelines, suggested by the Malaysian Food Regulations (30 mg/kg ww; [33]), and FAO [29] which were based in the countries of New Zealand, the UK, and Australia, with the range of the legal limits of Cu as 20–70 mg/kg ww.

The present Cu ranges (8.29–32.2 mg/kg dry weight; 1.41–5.47 mg/kg wet weight) were within those (2.82–103 mg/kg dw) reported by Yap et al. [44] in 40 populations of *P. viridis* from coastal waters of Peninsular Malaysia. The present Cu ranges were within those (23–35 mg/kg dw) reported for the coastal waters of Singapore [22], from India (0.36–1.63 mg/kg ww) [45], and from Peninsular Malaysia (6.31–20.10 mg/kg dw; [23]) but lower than those (8.90–130 mg/kg dw) from Hong Kong coastal waters [46]; de Astudillo et al. [47] reported the metal levels in *P. viridis* collected from Trinidad and Venezuela were Cu (1.02–3.43 mg/kg ww). Nicholson and Szefer [48] showed that Cu concentrations ranged from 10.1 to 18.0 mg/kg dw in the soft tissues of *P. viridis* in contaminated and unpolluted areas of Hong Kong coastal waters. Fung et al. [49] (2004) reported the concentrations (mg/kg dw) in *P. viridis* collected from Minjiang and Jiulongjiang (east coast of China) as Cu (5.56–11.1). Chinnadurai et al. [50] reported that the Cu concentrations (0.21–3.43 mg/kg ww) in *P. viridis* were collected from the Vembanad Estuary, Kerala (India). Aktar et al. [55] reported the *P. viridis* collected from Cox’s Bazar (Bangladesh) was 7.26 to 8.81 mg/kg dw for Cu. Kamaruzzaman et al. [56] reported Cu (8.96 mg/kg dw) in *P. viridis* collected from the Muar estuary. Mussels collected in 2009 from the Pekan River estuary (Pahang) coastal waters were reported as Cu (19.1 mg/kg dw) [57]. Mussels collected from Sungai Masai in the SOJ, Sheng et al. [58] reported that the concentration (mg/kg dw) was Cu (7.80). Mamat et al. [59] reported the *P. viridis* collected from KPP was 11.2–13.8 mg/kg dw for Cu. Soegianto et al. [60] reported that the mussels collected from the East Java coast contained Cu (0.07–4.42 mg/kg ww). Hence, the present Cu ranges are generally within those reported in the same mussel species in the literature.

#### 3.4.2. Comparisons of Copper Target Hazard Quotients

Values of Cu EDI and Cu THQ in the mussels are summarized in Table 3. After 10 weeks of depuration, the values of EDI decreased from 3.53 to 1.42 in KPP to KSM. The Cu EDI values decreased from 3.53 to 0.91 in KPP to SB. After 10 weeks of depuration, the values of THQ decreased from 0.09 to 0.04 in KPP to KSM. The THQ values decreased from 0.09 to 0.02 in KPP to SB. All the THQ values were below 1.00, indicating that there was no noncarcinogenic risk of Cu in the transplanted mussels to SB and KSM. In fact, the depuration transplantation study in both sites further reduced the Cu risk to consumers. The Cu human health risk was significantly (*p* < 0.05) lower after the mussels were transplanted to SB and KSM from KPP.

#### 3.4.3. Comparisons between Cu Estimated Weekly Intake (EWI) and Provisional Tolerable Weekly Intake (PTWI)

Values of Cu EWI in *P. viridis* and Cu PTWI in the mussels are summarized in Table 3. After 10 weeks of depuration, the values of Cu EWI decreased from 24.7 to 9.92 µg/week in KPP to KSM with a 59.8% reduction. In comparison to the Cu PTWI, the Cu EWI at week 0 contributed 0.011% and further reduced to 0.005% after 10 weeks of depuration. The Cu EWI values decreased from 24.7 to 6.37 µg/week in KPP to SB with 74.2%. In comparison to the Cu PTWI, the Cu EWI at week 0 contributed 0.011% and was further reduced to 0.003% after 10 weeks of depuration. Clearly, all of the EWI values were significantly lower than the Cu PTWI value (217,000 µg/week). Therefore, mussel consumption was not considered to have adverse effects of Cu on consumers based on the FAO/WHO JECFA guidelines [39,40].

According to research by Jovic and Stankovic [66], Croatia had the lowest Cu mean level (4.46 mg/kg ww), whereas Montenegro had the highest permissible weekly consumption of mussels (140 kg). In contrast, Jovic and Stankovic [66] found that, based on the mean Cu contents in mussels from the Adriatic Sea, the weekly intake of Cu was expected to range from 0.19 to 0.56 mg/person/week for heavy mollusk consumers and 1.12 mg/person/week.

All living things require numerous enzymes, which require the element Cu. It is necessary to synthesize hemoglobin [71] and a respiratory protein in mollusks containing Cu [72].

### 3.5. Fe

#### 3.5.1. Fe Safety Guidelines and Comparison with Reported Studies

After 10 weeks of transplantation, the percentages of Fe reductions were 78.8% and 51.3% from KPP to SB and KPP to KSM, respectively (Table 1 and Table 2; Figure 2).

The Fe in Figure 2 used an exponential decay model to estimate the best plot with a decay constant (λ) of 0.14 (R = 0.99; *p* < 0.05) to summarize the negative relationship between Ni levels and weeks after transplantation from KPP to KSM. Similarly, a higher decay constant (λ) of 0.24 (R = 0.78; *p* < 0.05) summarizes the negative relationship between Fe levels and weeks after transplantation from KPP to SB. This exponential regression can model a situation of faster decrement of Fe levels in the experimental transplanted KPP to SB than KPP to KSM.

The current Fe level at KPP (1876 mg/kg dw) was greater than those (105–1778 mg/kg dw) reported by Yap et al. [44] in 40 populations of *P. viridis* from the coastal waters of Peninsular Malaysia, those (70.83–80.55 mg/kg ww) reported for the coastal waters of Karnataka by Sasikumar et al. [45], and Hong Kong coastal waters (330–1280 mg/kg dw, Liu and Kueh [46]. Nicholson and Szefer [48] discovered that Fe concentrations were 354 mg/g dw in the soft tissues of *P. viridis* that were taken from a contaminated location in Kennedy Town, Hong Kong. Fung et al. [49] reported the concentrations (mg/kg dw) in *P. viridis* collected from Minjiang and Jiulongjiang (east coast of China) as Fe (271-1002). Chinnadurai et al. [50] reported that the Fe concentrations (55.5–98.5 mg/kg ww) in *P. viridis* collected from Vembanad Estuary, Kerala (India). Mussels collected in 2009 from the Pekan River estuary (Pahang) coastal waters were reported as Fe (576 mg/kg dw) [57]. Therefore, the current Fe ranges are, therefore, comparable to and within those found in *P. viridis* as described in the literature.

Up to 2015, there are scarcely any Fe maximum limits established by the WHO, FDA, FAO, or other nations. Prior to now, JECFA [34], based on the opinions of a global panel of experts, came to the conclusion that there was still some doubt regarding the maximum level of Fe that could be tolerated. As a result, it is impossible to compare MPLs with the available Fe data. However, there were hardly any Fe maximum limits set by WHO/FDA/FAO or other countries until 2015. Earlier, JECFA [34], based on the collective views of an international group of experts, concluded that there was still uncertainty regarding the Fe maximum level that can be tolerated. Therefore, a comparison with MPLs with the present Fe data is not possible. Despite the possibility that KPP might expose consumers to an Fe risk, the Fe risk of ingesting mussels from KPP remained unclear due to the small number of MPLs available for comparison.

#### 3.5.2. Comparisons of Fe Target Hazard Quotients

The values of Fe EDI and Fe THQ in the mussels are summarized in Table 3. After 10 weeks of depuration, the values of EDI decreased from 205 to 99.8 in KPP to KSM. The Fe EDI values decreased from 205 to 43.4 in KPP to SB. After 10 weeks of depuration, the values of Fe THQ decreased from 0.29 to 0.14 in KPP to KSM. The THQ values decreased from 0.29 to 0.06 in KPP to SB. All the Fe THQ values were below 1.00, indicating that there was no noncarcinogenic risk of Fe in the transplanted mussels to SB and KSM. In fact, the depuration transplantation study in both sites further reduced the Fe risk to consumers. The Fe human health risk was significantly (*p* < 0.05) lower after the mussels were transplanted to SB and KSM from KPP.

#### 3.5.3. Comparisons between Fe Estimated Weekly Intake (EWI) and Provisional Tolerable Weekly Intake (PTWI)

Values of Fe EWI in *P. viridis* and Fe PTWI in the mussels are summarized in Table 3. After 10 weeks of depuration, the values of Fe EWI decreased from 1434 to 698 µg/week in KPP to KSM with a 51.3% reduction. In comparison to the Fe PTWI, the Fe EWI at week 0 contributed 0.413% and further reduced to 0.201% after 10 weeks of depuration. The Fe EWI values decreased from 1434 to 304 µg/week in KPP to SB with a 78.8% reduction. In comparison to the Fe PTWI, the Fe EWI at week 0 contributed 0.413% and further reduced to 0.088% after 10 weeks of depuration. Clearly, all of the EWI values were significantly lower than the Fe PTWI value (347,200 µg/week). Therefore, mussel consumption was not considered to have adverse effects of Fe on consumers based on the FAO/WHO JECFA guidelines [34,40]. The Institute of Medicine [73] suggested 45 mg/day (or mg/kg bw/day) as the tolerated upper consumption level for Fe.

According to a research by Jovic and Stankovic [66], Croatia consumed the most mussels per week (10.5 kg; Fe mean: 32.1 mg/kg ww), and Albania consumed the fewest (2.52 kg; Fe mean: 133.5 mg/kg ww). Although other foods may increase the PTWI of Fe, these percentages do not raise much public concern because Fe is an essential component of living tissues. Consumers of high-level mollusks (0.250 kg) were predicted by Jovic and Stankovic [66] to ingest 33.4 mg Fe weekly.

In the form of hemoglobin, which carries oxygen in the blood, Fe supports vital bodily processes [34]. Generally speaking, Fe is not considered to be damaging to health until it is consumed at extremely high levels, and ongoing Fe iron excess may eventually affect the activity of the heart and liver [74].

### 3.6. Zn

#### 3.6.1. Zn Safety Guidelines and Comparison with Reported Studies

After 10 weeks of transplantation, the percentages of Zn reductions were 54.6% and 64.4% from KPP to SB, and KPP to KSM, respectively (Table 1 and Table 2; Figure 2).

The Zn in Figure 2 used an exponential decay model to estimate the best plot with a decay constant (λ) of 0.20 (R = 0.95; *p* < 0.05) to summarize the negative relationship between Zn levels and weeks after transplantation from KPP to KSM. Similarly, a lower decay constant (λ) of 0.13 (R = 0.77; *p* < 0.05) summarizes the negative relationship between Zn levels and weeks after transplantation from KPP to SB. This exponential regression can model a situation of a lower decrement of Zn levels in the experimental transplanted KPP to SB than in KPP to KSM.

The current Zn ranges (58.7–165 mg/kg dry weight; 9.98–28.1 mg/kg ww) were greater than those (50.9–138 mg/kg dw) reported by Yap et al. [44] in 40 populations of *P. viridis* from the coastal waters of Peninsular Malaysia. The current Zn ranges were within those reported in Peninsular Malaysia (53.82–128.90 mg/kg dw; [23]), in the area of Victoria Harbour in Hong Kong (21.0–109 mg/kg dw; [22]), and in the coastal waters of Singapore (185–446 mg/kg dw; [46]), but higher than those from the coastal waters of Karnataka, Southwest Coast of India; de Astudillo et al. [47] reported the metal levels in *P. viridis* collected from Trinidad and Venezuela were Zn (8.75–98.2 mg/kg ww). Nicholson and Szefer [48] showed that Zn concentrations ranged from 108 to 152 mg/kg dw in the soft tissues of *P. viridis* in contaminated and unpolluted areas of Hong Kong coastal waters. Fung et al. [49] reported the concentrations of *P. viridis* collected from Minjiang and Jiulongjiang (east coast of China) as Zn (62.8–231 mg/kg dw). Chinnadurai et al. [50] reported that the Zn concentrations (19.8–73.4 mg/kg ww) in *P. viridis* collected from the Vembanad Estuary, Kerala (India). Aktar et al. [55] reported the *P. viridis* collected from Cox’s Bazar (Bangladesh) was 28.12–33.82 mg/kg dw for Zn. Kamaruzzaman et al. [56] reported Zn (86.7 mg/kg dw) in *P. viridis* collected from the Muar estuary. Mussels collected in 2009 from the Pekan River estuary (Pahang) coastal waters were reported as Zn (45.5 mg/kg dw) [57]. In mussels collected from Sungai Masai in the SOJ, Sheng et al. [58] reported that the concentration was Zn (38.9 mg/kg dw). Soegianto et al. [60] reported that the mussels collected from the East Java coast contained Zn (0.16–8.10 mg/kg ww). The current Zn ranges are therefore equivalent to or within those found in *P. viridis* as reported in the literature.

For Zn, the comparisons were based on MPLs suggested by FAO [29], in the range of 40–150 mg/kg ww based on the countries New Zealand, the UK, and Australia, and the Malaysian Food Regulations (100 mg/kg ww; [33]). Hence, the present Zn levels in *P. viridis* were all well below the Zn safety guidelines. There was no evident Zn danger associated with eating mussels from the KPP.

#### 3.6.2. Comparisons of Zinc Target Hazard Quotients

Values of Zn EDI and Zn THQ in the mussels are summarized in Table 3. After 10 weeks of depuration, the values of EDI decreased from 18.1 to 6.44 in KPP to KSM. The Zn EDI values decreased from 18.1 to 8.21 in KPP to SB. After 10 weeks of depuration, the values of Zn THQ decreased from 0.06 to 0.02 in KPP to KSM. The Zn THQ values decreased from 0.06 to 0.03 in KPP to SB. All the THQ values were below 1.00, indicating that there was no noncarcinogenic risk of Zn in the transplanted mussels to SB and KSM. In fact, the depuration transplantation study in both sites further reduced the Zn risk to consumers. The Zn human health risk was significantly (*p* < 0.05) lower after the mussels were transplanted to SB and KSM from KPP.

#### 3.6.3. Comparisons between Zn Estimated Weekly Intake (EWI) and Provisional Tolerable Weekly Intake (PTWI)

The values of Zn EWI in *P. viridis* and Zn PTWI in the mussels are summarized in Table 3. After 10 weeks of depuration, the values of Zn EWI decreased from 127 to 45.1 µg/week in KPP to KSM with a 64.5% reduction. In comparison to the Zn PTWI, the Zn EWI at week 0 contributed 0.029% and further reduced to 0.010% after 10 weeks of depuration. The Zn EWI values decreased from 127 to 57.5 µg/week in KPP to SB with a 54.7% reduction. In comparison to the Zn PTWI, the Zn EWI at week 0 contributed 0.029% and further reduced to 0.013% after 10 weeks of depuration. Clearly, all of the EWI values were significantly lower than the Zn PTWI value (434,000 µg/week). Therefore, mussel consumption was not considered to have adverse effects of Zn on consumers based on the FAO/WHO JECFA guidelines [39,40]. 

The Institute of Medicine [73] suggested 40 mg/day (or mg/kg bw/day) as the tolerated maximum consumption threshold for Zn (upper limit for elemental Zn). The lowest number of mussels (6.68 kg per week) with the greatest Zn mean value (62.9 mg/kg ww) may be consumed in Croatia, and the largest amount (19.8 kg per week; Zn mean: 21.2 mg/kg ww) in Italy, according to Jovic and Stankovic [66]. In contrast, Jovic and Stankovic [66] found that based on the estimated Zn contents in mussels from the Adriatic Sea, the anticipated weekly intakes of Zn for frequent mollusk consumers were 7.86 mg/person/week and 15.7 mg/person/week, respectively.

Zn is one of the most crucial trace elements for metabolic activities in the human body, since it is required for cells and is an enzyme cofactor [39,75]. However, consuming too much dietary Zn can result in poisoning symptoms such as arteriosclerosis, pancreatic damage, and issues with protein metabolism [75,76]. Some specialists believe that long-term Zn and Cu exposure can cause Parkinson’s disease [72].

## 4. General Discussion

### 4.1. Lower Metal Levels Are Expected and Well-Supported by the Literature

When organisms from polluted areas were transplanted to clean areas, several authors also reported lower levels of metals after the transplantation period such as Zn in the *Mytilues edulis* [77], Cd and Cu in *Crassostrea gigas* [78], Cr, Cu and Zn in *Mercenaria mercenaria* [79], and Ag, Co and Ni in *Isognomon isognomon* [18].

Due to the depuration being reliant on the transplantation duration, the PTM levels could be far from reaching the concentrations detected in the resident population (W0) as compared to week 10’s levels [18]. This demonstrated that over the 10 weeks of *P. viridis*’ transplantation, the metals were not completely eliminated. According to previous research [80,81], the equilibration of trace metals in bivalves with their environment might take anywhere from 30 days to 77 days, and even longer for some other species, Due to past exposure histories, different life stages of bivalves, related metabolic activity with modifications to a new habitat, temperature variations, and food availability, different species may have varied equilibration durations [82].

The rate of depuration in the mussels then slowed down after two weeks, which may have been due to some firmly bound compartments, such as metallothionein and lysosomes for PTMs. Metallothionein is critical for the removal of PTMs in mussels, according to Viarengo et al. [83]. According to Phillips [9] and Rainbow [84], regulation and sequestration are crucial strategies for reducing the negative effects of high concentrations of these metals. The present field-based depuration study indicated that *P. viridis* was a good biomonitor of the bioavailability of metals, as indicated by *M. galloprovincialis* [85]. 

In a transplantation experiment, Cardoso et al. [12] investigated the bioaccumulation and depuration capacities of mercury by the edible bivalve *Scrobicularia plana* in a coastal lagoon (Ria de Aveiro, Portugal). During this brief absorption time, the transplanted organisms attained 20–30% of the quantities seen in resident contaminated species. After the exposure time, the organisms were moved to a clean environment for more than 25 days of depuration. The organisms lost about half of their body burden of mercury by the end of the transplantation period, and those from the least contaminated site nearly caught up to the concentrations observed in the reference area. Consequently, the finding implied that *S. plana* was a viable biomonitoring species since it collects the pollutant to a significant amount quickly and has a poor metal retention capacity (short biological half-life) when exposed to clean sediments.

According to Zimmer et al. [17], mussels *M. edulis* transplanted from the contaminated Ivittuut mining site to the pristine Kugnait Bay only released 7–21% of their initial Pb content, and this release only occurred within the first 10 days of transplantation. Thereafter, the Pb content remained constant. Measurements of Hg and As absorption and depuration rates were made using caged mussels in transplant studies carried out by Hickey et al. [86]. The Hg absorption and depuration half-lives in mussels were 6 to 12 months. According to Odzak et al. [87], Zn levels in transplanted *M. galloprovincialis* did not fluctuate according to the seasons, most likely because of the presence of a control mechanism.

The 10 weeks of depuration of PTMs to the clean sites have evidently proven to have reduced consumers’ health risks, with significantly (*p* < 0.05) lower EWI values, of *P. viridis*. Given that PTMs enter the human body from other sources, notably through other meals, and since the limit, in this case, is readily attainable, this information warrants additional attention. After 10 weeks, the depurations of the mussels to KSM and SB from KPP have less of an effect on consumer health in terms of Ni concentrations. Given that Cd enters the body from other sources, notably through other meals, and that the limit, in this case, is quickly achieved, this information warrants closer attention. This demonstrated that Pb enters the human body mostly through the soft tissue of *P. viridis*. If one considers that Pb also enters the human body from other sources/foods, these facts become much more concerning. Yap et al. [88] reported that boiling the soft tissues for 15 min could significantly (*p* < 0.05) reduce the Zn concentrations after the boiling process. Therefore, boiling can further reduce the risk of Zn toxicity to consumers.

### 4.2. Seafood–Energy–Water Nexus: A Food Safety Concern in the Straits of Johore

**Figure 3:**
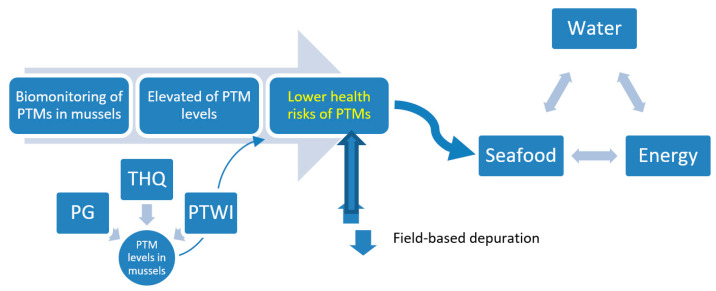
Overall connection of the lower human health risks of potentially toxic metals (PTM)s in the depurated mussels in the Straits of Johore under of the seafood–energy–water Nexus. Note: PG = permissible guidelines; THQ = target hazard quotient; PTWI = provisional tolerable weekly intake.

The overall connection of the lower human health risks of PTMs in the depurated mussels under of the seafood–energy–water Nexus is presented in Figure 3. Due to the exponential increase in energy and water demand that is associated with this worldwide transformation, concerns over resource security have increased [89]. Continuous evaluation of the PTMs’ potential dangers to human health in the aquaculture-farmed mussels in the SOJ is necessary for mitigation strategies to lessen the severity of the depletion and its environmental implications. This qualifies as a “Nexus” and can be recognised as such. This study concentrated on the safety of mussels, which might be a key factor in the supply chain for sustainable and high-quality seafood products. It also considered biomonitoring and health risk assessments of PTMs of the mussel resources along the chain (Figure 3). This knowledge can help the mussel seafood-industry supply chain attain sustainability via cogent policymaking and management [89].

The main type of aquaculture used in interior regions is coastal aquaculture, where pollution is a major problem. The risk assessment approach suggested by Bai et al. [90] should be expanded for aquaculture applications that need to monitor more factors to protect aquaculture quality and support efficient safety risk management. The high bioaccumulation of contaminants in marine aquacultural mussels is caused by the presence of pollutants, either in the closed-water habitat or in the constantly injected water supply [21]. Organic and inorganic contaminants, including potentially harmful metals, may be present in the agricultural water used in coastal areas [6,21]. These pollutants can all bioaccumulate in mussel tissues and enter them. The food safety objective approach is consistent with the core principles of process validation, according to Keener [91]. Food safety proof is extremely difficult, if not impossible. The success of a preventive measure for lowering a defined danger to an adequate level or concentration that is consistent with accomplishing public health objectives, however, may be confirmed with a high degree of statistical confidence when the right instruments and procedures are used. All of the aforementioned data point out that mussel seafood product quality should be regularly monitored for PTM contamination with particular reference to human health risk assessments of PTMs in the semienclosed SOJ, which is a hotspot anthropogenic source [21,23,24,25] with high aquacultural commercial mussels since many decades ago.

Abisha et al.’s [92] depictions of the different abiotic stress factors that contribute to the abrupt climate change included adaptation strategies used in different aquaculture sources, including freshwater, inland saline water, brackish water, coastal waters, and culture-based capture fisheries, as well as the consequences of these strategies in the future. The long-term linkages between water, food, and energy are crucial for the growth and protection of the economy and the environment. Food, energy, and water are essential economic inputs and a requirement for economic development [93]. One significant component of the marine fisheries’ carbon sink is the cultivation of shellfish and algae [94]. Food, energy, and water are crucial resources for human survival and national growth, but competition has risen between the three, particularly in coastal areas. The majority of research to far has focused on land [95]. In the coastal regions of China, Zhu et al. [95] investigated the impact of marine resources on easing the pressure of the food-energy-water nexus. In sum, the depuration technique of the mussels from the polluted waters to clean coastal waters supports regional sustainable development and advance a more thorough understanding of and protection for the coastal waters.

## 5. Conclusions

With the transplantation of *P. viridis* from KPP, a polluted site, to KSM and SB, two unpolluted sites in the SOJ, the current field study sought to measure the levels of six PTMs. Interestingly, after 10 weeks of depuration in the clean sites, the six PTMs in SB and KSM showed decreases of 55.6–88.4% and 51.3–91.7%., respectively. After 10 weeks of depuration of the transplanted polluted mussels to the two unpolluted sites in the SOJ, the present findings of significantly (*p* < 0.05) lower levels of safety guidelines, significantly (*p* < 0.05) lower values of THQ, and significantly (*p* < 0.05) lower values of EWI (when compared to PTWI) of all the six PTMs were recorded. Hence, the PTMs’ noncarcinogenic risks to consumers are further diminished. This depuration method might be advised from an aquacultural perspective to lessen consumer health concerns associated with PTMs.

## Figures and Tables

**Table 1:** Overall statistics of concentrations (mg/kg dry weight) of 6 potential toxic metals in the soft tissues of *Perna viridis* during the transplantation study from Kg. Pasir Puteh to Sungai Belungkor (P-B) and to Kg. Sungai Melayu (P-M).

P-B	Cd	Cu	Ni	Fe	Pb	Zn
Minimum	1.87	8.29	5.05	395	1.48	74.89
Maximum	4.74	32.16	35.77	1867	12.74	164.92
Mean	2.67	14.66	19.07	775	4.95	99.98
Median	2.02	9.09	17.73	419	2.80	80.06
Std Error	0.69	5.84	6.37	364	2.62	21.70
P-M	Cd	Cu	Ni	Fe	Pb	Zn
Minimum	1.92	12.92	10.51	909	1.06	58.68
Maximum	4.74	32.16	35.77	1867	12.74	164.92
Mean	3.29	23.99	20.40	1384	6.97	122.11
Median	3.25	25.43	17.66	1381	7.05	132.43
Std Error	0.59	4.12	5.45	206	2.41	22.94

**Table 2:** Percentages (%) of reduction of 6 potentially toxic metals in the soft tissues of *Perna viridis* after weeks of transplantation from Kg. Pasir Puteh to Sungai Belungkor (Puteh to Belungkor) and to Kg. Sungai Melayu (Puteh to Melayu).

Puteh to Belungkor	Cd	Cu	Ni	Fe	Pb	Zn
Week 2	56.74	69.51	44.67	76.57	75.93	50.02
Week 6	58.23	73.97	56.19	78.53	80.21	52.90
Week 10	59.70	74.22	85.88	78.81	88.35	54.59
Puteh to Melayu	Cd	Cu	Ni	Fe	Pb	Zn
Week 2	26.16	13.96	45.09	17.38	37.66	13.01
Week 6	36.92	27.89	56.14	34.69	51.71	26.40
Week 10	59.49	59.83	70.62	51.29	91.68	64.42

**Table 3:** Values of estimated daily intake (EDI, µg/kg body weight/day), target hazard quotient (THQ), and estimated weekly intake (EWI, µg/kg body weight/day) for 6 potentially toxic metals in the total soft tissues of *Perna viridis* transplanted from Kg. Pasir Puteh to Kg. Sungai Melayu (Puteh to Melayu) and to Sungai Belungkor (Puteh to Belungkor) from the present study.

Puteh to Melayu		Cd			Cu			Ni			Fe			Pb			Zn	
PTWI			361.5			217,000			5642			347,200			1302			434,000
Week	EDI	THQ	EWI	EDI	THQ	EWI	EDI	THQ	EWI	EDI	THQ	EWI	EDI	THQ	EWI	EDI	THQ	EWI
0	0.52	0.52	3.64	3.53	0.09	24.7	3.92	0.20	27.5	205	0.29	1434	1.40	0.399	9.78	18.1	0.06	127
2	0.38	0.38	2.69	3.04	0.08	21.2	2.15	0.11	15.1	169	0.24	1185	0.87	0.249	6.10	15.7	0.05	110
6	0.33	0.33	2.30	2.54	0.06	17.8	1.72	0.09	12.0	134	0.19	936	0.68	0.193	4.72	13.3	0.04	93.2
10	0.21	0.21	1.47	1.42	0.04	9.92	1.15	0.06	8.07	99.8	0.14	698	0.12	0.033	0.81	6.44	0.02	45.1
Puteh to Belungkor		Cd			Cu			Ni			Fe			Pb			Zn	
PTWI			361.5			217,000			5642			347,200			1302			434,000
Week	EDI	THQ	EWI	EDI	THQ	EWI	EDI	THQ	EWI	EDI	THQ	EWI	EDI	THQ	EWI	EDI	THQ	EWI
0	0.52	0.52	3.64	3.53	0.09	24.7	3.92	0.20	27.5	205	0.29	1434	1.40	0.399	9.78	18.1	0.06	127
2	0.23	0.23	1.57	1.08	0.03	7.53	2.17	0.11	15.2	48.0	0.07	336	0.34	0.096	2.35	9.04	0.03	63.3
6	0.22	0.22	1.52	0.92	0.02	6.43	1.72	0.09	12.0	44.0	0.06	308	0.28	0.079	1.94	8.52	0.03	59.6
10	0.21	0.21	1.47	0.91	0.02	6.37	0.55	0.03	3.88	43.4	0.06	304	0.16	0.046	1.14	8.21	0.03	57.5

## Data Availability

The data presented in this study are available on request from the corresponding author.

## Supplementary Materials

The following supporting information can be downloaded at: https://www.mdpi.com/article/10.3390/foods12101964/s1, Table S1: Variation of shell length (cm), shell width (cm), shell height (cm), and growth rate (cm/week); Table S2: A comparison of trace metal concentrations (mean ± SE, µg/kg dry weight) between measured values and certified values in the certified reference materials (CRM) for mussel tissue (NIST 2976), dogfish liver (DOLT-3, National Research Council Canada); Table S3: Values of oral reference dose (ORD, µg/kg body weight/day) and provisional tolerable weekly intakes (PTWI, mg/kg body weight/week) in the six potentially toxic metals used in the present study.
