# Supplementary material for: Lower Health Risks of Potentially Toxic Metals after Transplantation of Aquacultural Farmed Mussels from a Polluted Site to Unpolluted Sites: A Biomonitoring Study in the Straits of Johore
Source: Foods. 2023 May 11;12(10):1964. doi: 10.3390/foods12101964 (PMC10217254; doi:10.3390/foods12101964)
Supplement: Supplementary file 1 [file foods-12-01964-s001.zip › foods-2367761-supplementary.pdf]

Table S1. Variation of shell length (cm), shell width (cm), shell height (cm), and growth rate (cm/week).

	Shell Length						Growth rate	
**Week**	Sg Melayu			Belungkor			Sg Melayu	Belungkor
**0**	30.45	±	2.64	34.46	±	1.33	NA	
2	53.17	±	3.14	55.17	±	2.37	11.36	10.36
6	66.15	±	4.57	67.49	±	1.81	3.25	3.08
10	78.74	±	5.39	81.26	±	4.86	3.15	3.44
	Shell Width						Growth rate	
Week	Sg Melayu			Belungkor			Sg Melayu	Belungkor
0	9.07	±	1.04	10.22	±	1.26	NA	
2	16.96	±	3.11	17.28	±	2.67	3.95	3.53
6	19.37	±	4.31	20.09	±	0.66	0.60	0.70
10	23.41	±	3.94	24.09	±	0.66	1.01	1.00
	Shell Height						Growth rate	
Week	Sg Melayu			Belungkor			Sg Melayu	Belungkor
0	14.64	±	1.54	15.66	±	2.37	NA	
2	22.39	±	3.88	23.47	±	3.19	3.88	3.91
6	29.61	±	4.32	30.66	±	0.68	1.81	1.80
10	35.10	±	4.16	35.66	±	0.68	1.37	1.25

Table S2. A comparison of trace metal concentrations (mean ± SE, μg/kg dry weight) between measured values and certified values in the Certified Reference Materials (CRM) for mussel tissue (NIST 2976), Dogfish Liver (DOLT-3, National Research Council Canada).

**Metals**	**Certified values (C)**	**Measured value (M)**	**Percentage of recovery [(M/C) x 100]**
	Mean	Mean	
Cu	31.2 ± 1.00	26.8 ± 0.25	85.93
Cd	19.4 ± 0.60	14.7 ± 0.34	75.67
Fe	1484 ± 57.0	1213 ± 10.7	81.77
Ni	2.72 ± 0.35	3.37 ± 0.20	123.90
Pb	37.4 ± 12.8	29.4 ± 0.33	78.56
Zn	86.6 ± 2.40	76.1 ± 0.81	99.90

Table S3. Values of oral reference dose (ORD, μg/kg body weight/day), and provisional tolerable weekly intakes (PTWI, mg/kg body weight/week) in the six potentially toxic metals used in the present study.

**Metal**	**ORD***	**Intake**	**Reference**	**PTWI**	**PTWI for 62 kg adult**
Zn	300	PTDI (mg/kg bw/day) of Zn was calculated from a PMTDI of 1.00 mg/kg BW/day	JECFA (1982, 2021)	1.00 mg/kg BW/day) x 7 days= 7.00 mg/kg BW/week	7.00 mg/kg BW/week x 62 kg= 434 mg/week (434000 μg/week).
Fe	700	A PMTDI of 0.80 mg/kg BW/day	JECFA (1983, 2021)	0.80 mg/kg BW/day) x 7 days= 5.60 mg/kg BW/week	5.60 mg/kg BW/week x 62 kg= 347.2 mg/week (347200 μg/week).
Cu	40.0	A PMTDI of 0.50 mg/kg BW/day	JECFA (1982, 2021)	0.50 mg/kg BW/day) x 7 days= 3.50 mg/kg BW/week.	3.50 mg/kg BW/week x 62 kg= 217 mg/week (217000 μg/week).
Ni	20.0	A TDI of 13 μg/kg BW	EFSA (2020)	13 μg/kg BW x 7 days= 91 μg/kg BW	91 μg/kg BW x 62 kg= 5642 μg/week
Pb	3.50	For adults, the TDI (μg/kg bw/day) of Pb was calculated from the higher end of the range (3 μg/kg BW/day).	JECFA (2011)	3.00 μg/kg BW/day) x 7 days= 21.0 μg/kg BW/week	21.0 μg/kg BW/week x 62 kg= 1302 μg/week.
Cd	1.00	A PTMI of 25.0 μg/kg BW based on a month of 30 day. Therefore, PTDI= (25 μg/kg BW/month)/ 30 days= 0.833 μg/kg BW/day	JECFA (2011, 2021)	0.833 μg/kg BW/day) x 7 days= 5.83 μg/kg BW/week	5.83 μg/kg BW/week x 62 kg= 361.5 μg/week

Note: *= The values of oral reference dose (ORD) for all metals are specified by the US EPA regional screening level (USEPA, 2021). BW= Body weight. TDI= Tolerable daily intake; PTMI= provisional tolerable monthly intake; PTDI= provisional tolerable daily intake. PMTDI= provisional maximum tolerable daily intake.
